# The inv dup (15) or idic (15) syndrome (Tetrasomy 15q)

**DOI:** 10.1186/1750-1172-3-30

**Published:** 2008-11-19

**Authors:** Agatino Battaglia

**Affiliations:** 1 Stella Maris Clinical Research Institute for Child and Adolescent Neurology and Psychiatry, Calambrone, Pisa, Italy

## Abstract

The inv dup(15) or idic(15) syndrome displays distinctive clinical findings represented by early central hypotonia, developmental delay and intellectual disability, epilepsy, and autistic behaviour. Incidence at birth is estimated at 1 in 30,000 with a sex ratio of almost 1:1. Developmental delay and intellectual disability affect all individuals with inv dup(15) and are usually moderate to profound. Expressive language is absent or very poor and often echolalic. Comprehension is very limited and contextual. Intention to communicate is absent or very limited. The distinct behavioral disorder shown by children and adolescents has been widely described as autistic or autistic-like. Epilepsy with a wide variety of seizure types can occur in these individuals, with onset between 6 months and 9 years. Various EEG abnormalities have been described. Muscle hypotonia is observed in almost all individuals, associated, in most of them, with joint hyperextensibility and drooling. Facial dysmorphic features are absent or subtle, and major malformations are rare. Feeding difficulties are reported in the newborn period.

Chromosome region 15q11q13, known for its instability, is highly susceptible to clinically relevant genomic rearrangements, such as supernumerary marker chromosomes formed by the inverted duplication of proximal chromosome 15. Inv dup(15) results in tetrasomy 15p and partial tetrasomy 15q. The large rearrangements, containing the Prader-Willi/Angelman syndrome critical region (PWS/ASCR), are responsible for the inv dup(15) or idic(15) syndrome. Diagnosis is achieved by standard cytogenetics and FISH analysis, using probes both from proximal chromosome 15 and from the PWS/ASCR. Microsatellite analysis on parental DNA or methylation analysis on the proband DNA, are also needed to detect the parent-of-origin of the inv dup(15) chromosome. Array CGH has been shown to provide a powerful approach for identifying and detecting the extent of the duplication. The possible occurrence of double supernumerary isodicentric chromosomes derived from chromosome 15, resulting in partial hexasomy of the maternally inherited PWS/ASCR, should be considered in the differential diagnosis. Large idic(15) are nearly always sporadic. Antenatal diagnosis is possible. Management of inv dup(15) includes a comprehensive neurophysiologic and developmental evaluation. Survival is not significantly reduced.

The inv dup(15) or idic(15) syndrome can also be termed "tetrasomy 15q". About 160 patients have been reported in the medical literature [1-5].

## Background

The inv dup(15) or idic(15) syndrome (inverted duplication of proximal chromosome 15 or isodicentric 15 chromosome) displays distinctive clinical findings represented by early central hypotonia, developmental delay and intellectual disability, epilepsy, and autistic behavior. The latter is characterized by lack of social interaction, non-functional use of objects, primordial type of exploration, stereotypies, absent or very poor echolalic language, limited comprehension, and poor intention to communicate. Physically, there are only minor anomalies. Altogether, this picture constitutes a distinct and recognizable neurogenetic disorder, that can be suspected clinically, even before the cytogenetic confirmation.

Many rearrangements may occur in the imprinted chromosome region 15q11q13, which is known for its instability [6] due to the presence of repeated DNA elements [7,8]. The rearrangements include deletions associated either with Angelman syndrome (AS) or with Prader-Willi syndrome (PWS), according to parental origin [9]; translocations, inversions and supernumerary marker chromosomes formed by the inverted duplication of proximal chromosome 15. Interstitial duplications, triplications and balanced reciprocal translocations are much less frequent [10]. The inv dup (15) or idic (15) is the most common of the heterogeneous group of the extra structurally abnormal chromosomes (ESACs). Two cytogenetic types of inv dup (15) marker chromosomes have been identified, with different phenotypic consequences [11-14]. One is a metacentric or submetacentric and heterochromatic chromosome, smaller or similar to a G group chromosome, not containing the PWS/AS critical region (PWS/ASCR), and the cytogenetic description is dic(15)(q11). dic(15)(q11) can be familial or *de novo*, and is the most common ESAC accounting for about 70% of all small extra marker chromosomes. Most children with this aberration show a normal phenotype [15], although exceptions have been recorded [16]. The second type of inv dup (15) is as large as, or larger than, a G group chromosome and has 15q euchromatin. It includes the PWS/ASCR [17,18], and the cytogenetic description is dic(15)(q12 or q13). The vast majority of dic(15)(q12 or q13) derives from the two homologous maternal chromosomes at meiosis, and is reportedly associated with increased mean maternal age at conception, similar to other aneuploides. The presence of large inv dup (15) results in tetrasomy 15p and partial tetrasomy 15q. However, considerable structure heterogeneity has recently been described in a few patients [19]. This condition is associated with an abnormal phenotype, which constitutes the idic(15) syndrome [20,21]. In all such cases, standard cytogenetics must be associated with FISH (fluorescence *in situ *hybridization) analysis, using probes both from proximal chromosome 15 and from the PWS/ASCR [2,22]. Molecular studies, such as microsatellite analysis on parental DNA or methylation analysis on the proband DNA, are also needed in order to detect the parent-of-origin of the inv dup (15) chromosome [2,22]. Recently, array CGH (array comparative genomic hybridization) has been shown to provide a powerful approach to detect the duplication and its extent [4].

Maternally derived cytogenetic mosaicism with a normal cell line has been described in a small subset of individuals [23,24]. Recently, one patient with a mosaic paternally derived inv dup(15), showing a mild PWS phenotype, has been reported by Saitoh *et al*. [25].

## Epidemiology

Although poorly known, incidence at birth is estimated to be 1 in 30,000 with a sex ratio of almost 1 [3]. However, a higher incidence is plausible due to under-ascertainment. Since dysmorphic features are absent or subtle and major malformations are rare, chromosome analysis may not be thought to be indicated, and some individuals, particularly in the older age groups, probably remain undiagnosed.

## Clinical description

Developmental delay and intellectual disability affect all individuals with inv dup (15) and are usually moderate to profound [1,2,13,17,21,26,27]. Age of acquisition of motor milestones are seldom reported in the medical literature. However, it seems that sitting is achieved between 10 and 20 months of age, and walking between 2 and 3 years [17,21]. Expressive language is absent or very poor; it is often echolalic with immediate and delayed echolalia and pronoun reversal. Comprehension is very limited, contextual and accompanied by the gesture. Intention to communicate is absent or very poor [21,27]. The distinct behavior disorder shown by children and adolescents has been widely described as autistic or autistic-like. A strong association between autistic features and isodicentric chromosome 15 [idic (15)] was reported by Rineer *et al*. [28]. The patients reported by Borgatti *et al*. [29] met the clinical criteria for the diagnosis of autistic disorder by DSM IV (Diagnostic and Statistical Manual – Revision 4). Usually, these kids have gaze avoidance from very early on; shun body contact; stare at people as looking through them. They can be fascinated by certain sounds, by the water, or by spinning or any glittering objects. They usually prefer being left alone, lying on the back just looking at their fingers and taking bizarre postures. Symbolic play is usually never acquired. They show no interest toward their peers, and usually do not develop appropriate social interactions. When thwarted, they can react with outbursts of shouting or with aggressiveness. Non-functional use of objects with a primordial type of exploration is also observed. Stereotypies are frequently seen, including hand flapping, hand-wringing, hand-clapping over plane surfaces, finger biting, head turning, spinning him/herself for long periods of time. Hyperactivity has been reported in a number of cases [21,26,27].

Epilepsy with a wide variety of seizures can occur in these individuals, with onset between ages 6 months and 9 years [2,13,21,26,30]. Infantile spasms associated with an hypsarrhythmic EEG (electroencephalogram) have been reported in several patients [2,13,17,30]. Typical Lennox-Gastaut syndrome or Lennox-Gastaut-like syndrome was observed in the four inv dup (15) patients reported by Battaglia *et al*. [21]. These had tonic/atonic (as head drops or drop attacks), tonic-clonic seizures and atypical absences with onset between 4 and 8 years of age. Seizures were difficult to control in all, despite adequate antiepileptic treatment. Difficult to control seizures, associated with some degree of deterioration were also reported by other authors [13,17,26,31]. Complex partial and myoclonic seizures were observed in a number of other individuals [26]. Drug-resistant myoclonic absence-like seizures induced by emotionally gratifying stimuli (kissing, viewing of pleasant or funny events) have been reported in a 9-year-old boy [32]. A mild, adult-onset, generalized epilepsy with absence seizures and occasional head drops, and generalized tonic-clonic seizures has also been occasionally reported, with a good outcome [33]. Benign epilepsy with centro-temporal spikes with a benign evolution has been observed in another inv dup(15) individual [34].

**Figure 1:**
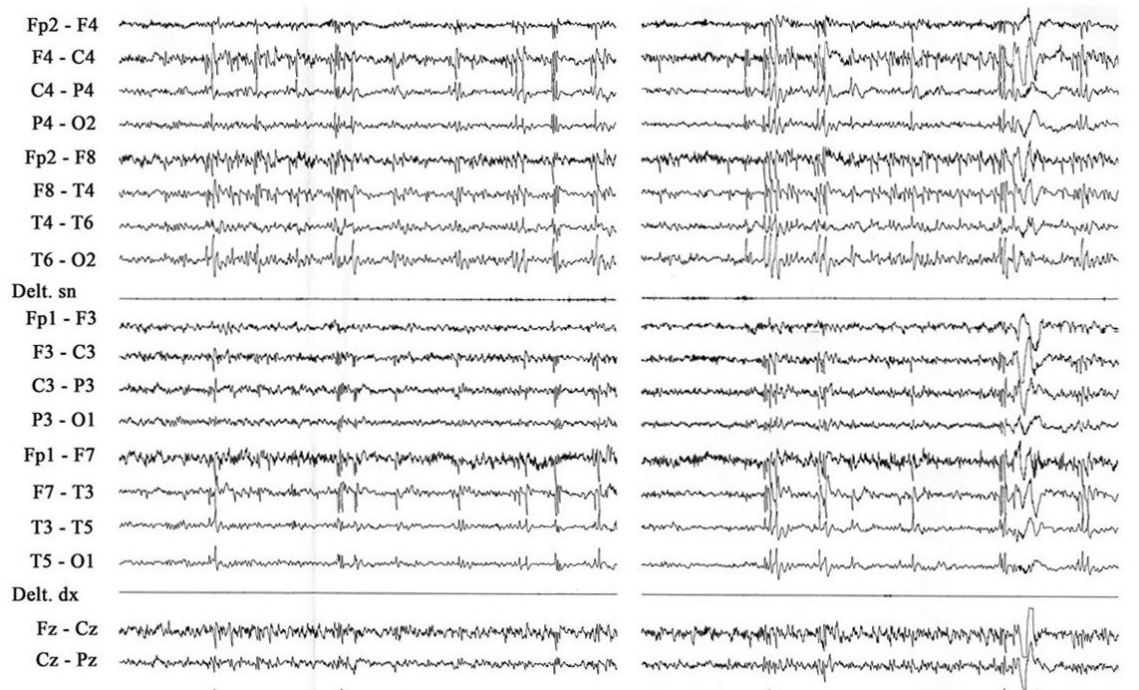
**Inv dup(15) or idic(15) patient at age 12 ys7 m**. Slow wave sleep. EEG showing diffuse spikes, polispikes, ill-defined polispike/wave complexes, somewhat predominant over the right hemisphere. Amplitude 100 **μ**V/cm; speed 1 sec/1.5 cm.

In the medical literature, very little is reported on the EEG characteristics in inv dup (15) syndrome, mainly due to the poor description of these findings and a total lack of serial EEG studies [2,13,26,27,30]. In 1997, Battaglia *et al*. [21] described in detail the EEG abnormalities observed in four such individuals. All received serial polygraphic-video-EEG recordings during wakefulness and sleep (overnight sleep in 2 of the 4) over a long period of time. All EEGs were abnormal, showing: 1) slow background activity; 2) absence or poverty of the rhythmic activities usually elicited over the posterior third of the brain, on eye closure; 3) multifocal discharges with variable hemispheric predominance; 4) frequent, large amplitude, generalized paroxysms, lasting 2 to 20 seconds, characterized by slow sharp element-spike/wave complexes, mostly accompanied by atypical absences; 5) frequent generalized bursts of fast rhythms during slow wave sleep in 2 of the 4 patients, accompanied by tachypnea and/or by an upward rotation of the eyes, and/or by tonic fits; 6) disruption of the usual sleep structure. Other EEG findings reported in the literature include generalized rhythmic 3.5–4 Hz spike-and-wave discharges, lasting 4–6 seconds [33]. Additional EEG abnormalities consist of diffuse spikes, polispikes, and ill-defined polispike/wave complexes, with variable hemispheric predominance (Fig. 1) [personal observation].

Muscle hypotonia is observed in almost all individuals, associated, in most of them, with joint hyperextensibility and drooling. Other physical findings are rather unspecific and may include: minor facial dysmorphisms, characterized by downslanting palpebral fissures, epicanthal folds, deep-set eyes, low-set and/or posteriorly rotated ears, highly arched palate, broad nose, anteverted nares; 5^th ^finger clinodactyly and unusual dermatoglyphics; partial 2^nd^–3^rd ^toe syndactyly [3,21]. Brachycephaly, frontal bossing, synophrys, broad nose, short philtrum, cleft palate, prominent mandible in adults, brachydactyly, and areas of increased and reduced skin pigmentation can be occasionally observed. Major malformations, such as ventricular septal defects, tetralogy of Fallot, unilateral renal agenesis, umbilical and inguinal hernias, hypospadias, cryptorchidism, and talipes seem to be rare [13,17,18,21,35-38]. Feeding difficulties are reported in the newborn period [5]. Growth is retarded in about 20%–30% of the patients. Microcephaly is observed in less than 20% of individuals, while macrocephaly in less than 3%. Hypogonadism is reported in about 20% [1,3]. Although puberty is reportedly normal in most individuals [39], pubertal disorders, such as central precocious puberty or ovarian dysgenesis, have been observed in three girls [40]. To the best of our knowledge, no individual with idic(15) syndrome is known to have reproduced.

Pregnancies and birth weights are mostly normal. Mean parental/maternal ages at birth of the probands were markedly advanced [2,41]. Brain neuroimaging (CT/MRI) does not show abnormal findings in most cases [21,29].

## Molecular characterization

Various genetic mechanisms have been hypothesized to explain clinical heterogeneity, including the size of chromosomal duplication, dosage effect of genes in this region, and the imprinting mechanism.

**Figure 2:**
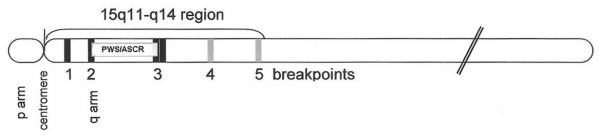
Schematic representation of chromosome 15, showing the five recurrent breakpoints (BP), and the ~4 Mb segment that encompasses the PWS/AS critical region.

The fact that tetrasomy of the PWS/ASCR is associated with a more severe phenotype than the one observed in trisomy, suggests that there is a dosage effect for a gene or genes in this region [5,42]. Furthermore, gene expression in this critical region is regulated by an imprinting mechanism [43]. Since only maternally inherited aberrations of chromosome 15q11-13 seem to be pathogenic, with the sole exception of one patient [44], it is likely that maternal genes, contained in this genomic region, act in a dosage-dependent manner and that their copy number is critical for normal brain development and function. Five recurrent breakpoints (BP) have been described in most cases and most idic(15) chromosomes arise through BP3:BP3 or BP4:BP5 recombination events (Fig. 2). The ~4Mb segment that encompasses the PWS/ASCR lies between BP2 and BP3. The most frequent form of idic (15) is characterized by an asymmetric recombination event between BP4 and BP5. This leads to tetrasomy for the interval from the centromere to BP4 and to trisomy from BP4 to BP5 [4]. Amongst the genes that are known to be located at or near the PWS/ASCR, an interesting relationship has been shown between the *α5 *and *β3 *GABA (gamma-aminobutyric acid) receptor subunit genes and the *P *gene. In fact, even though these genes are not imprinted, a deletion of both P alleles causes, in the mouse, rearrangements of α5 and β3 receptors producing a particular phenotype characterized by seizures, jerky gait and ataxia [45]. Tetrasomy of these genes, as seen in inv dup (15) syndrome, may alter the GABA receptor activity, upon which the major CNS (central nervous system) inhibitory mechanisms rely. This alteration could represent the biological basis for some clinical manifestations of the inv dup (15) syndrome individuals, such as seizures, hyperactivity, aggressiveness, and autistic disorder. Of interest, linkage disequilibrium between a marker in the **γ**-aminobutiric acid_A _receptor subunit gene, *GABRB3 *155CA-2, and autistic disorder has been reported by Cook *et al*. [46]. An additional gene, located more distally, the *SLC12A6 *(solute carrier, family 12, member 6), coding for a cation chloride cotransporter and expressed in the brain, heart, skeletal muscle, and kidney, could also potentially be implicated in the pathogenesis of seizures [47].

A phenotype-genotype correlation has been attempted, but it was problematic. In fact, component manifestations such as seizure history, behaviour disorder, and developmental status are difficult to assess accurately, especially in young children, and there is also considerable variation in the breakpoints [5, personal observation].

A recent study performed on post-mortem brain tissue from two individuals with 15q11-13 hexasomy and 15q11-13 tetrasomy respectively, suggests that genetic copy number variation, combined with additional genetic or environmental influences on epigenetic mechanisms, impact clinical heterogeneity and outcome of idic(15) syndrome [48].

## Diagnosis and diagnostic methods

Clinicians should suspect this syndrome in any infant or child with early central hypotonia, minor dysmorphic features, developmental delay/intellectual disability, autism or autistic-like behavior, who subsequently develops hard to control seizures/epilepsy (Table 1).

**Table 1:** Frequency of the main clinical characteristics of inv dup(15) or idic(15) syndrome

**Exceeding 75%**	**From 25% to 50%**	**Below 25%**
Hypotonia, lax ligaments	Brain abnormalities	Congenital heart defects

Developmental delay/Intellectual disability	Genitourinary tract defects	Microcephaly

Autistic behavior	Growth retardation	

Epilepsy		

Minor dysmorphic features (mainly involving the face)		

Standard cytogenetics (G-C-banding methods) must be associated with FISH analysis, using probes both from proximal chromosome 15 and from the PWS/ASCR [2,22]. Molecular studies, such as microsatellite analysis on parental DNA or methylation analysis on the proband DNA, are also needed in order to detect the parent-of-origin of the inv dup (15) chromosome [2,22]. Recently, array CGH has been shown to provide a powerful approach to detect the duplication and its extent [4].

## Differential diagnosis

Occurrence of double supernumerary isodicentric chromosomes derived from 15, resulting in partial hexasomy of the maternally inherited PWS/ASCR, has also been reported [4,49]. This disorder is, as well, associated with an abnormal phenotype, including severe psychomotor delay, hypotonia, joint hyperlaxity, brachycephaly, facial dysmorphisms, and small hands and feet [38].

Hypotonia in infancy can be severe and may lead to genetic evaluation for PWS, showing the idic(15) chromosome [21].

## Genetic counseling

Large SMC(15) (supernumerary marker chromosome), which include PWS/ASCR, or idic(15), are nearly always sporadic.

## Antenatal diagnosis

Prenatal diagnosis is possible, and has frequently been reported [50].

Cells obtained by chorionic villus sampling (CVS) at approximately 12 weeks' gestation, or amniocentesis, usually performed at approximately 15–18 weeks' gestation, can be analyzed by a combination of cytogenetic (G-C-banding, FISH), and molecular (methylation analysis) methods.

## Management

The management of inv dup (15) includes a comprehensive neurophysiologic and developmental evaluation.

A waking/sleeping polygraphic-video-EEG study is recommended in infancy and childhood in order to achieve the best characterization of the seizures. This is of the utmost importance for the first choice pharmacotherapy. In the author's experience, the most helpful drug treatment is represented by the association of valproate (VPA), and/or carbamazepine (CBZ) with lamotrigine (LTG). VPA is the first choice drug in those cases in whom the onset seizures are represented by atypical absences, whereas CBZ can be chosen whenever tonic seizures are the onset ones and the most frequent. Regular follow-up of seizure status and pharmacotherapy is essential.

A thorough developmental evaluation by a specialist should be performed at intervals appropriate for planning of early intervention, including physical, occupational and speech therapies, and for educational and vocational planning.

## Prognosis

Survival is not significantly reduced. However, very recently there has been the sudden, unexpected, and as yet unexplained death of six seemingly healthy young individuals with chromosome 15q duplication syndrome. Research is underway to investigate this phenomenon [51].

A global evolution of the adaptive behavior, social interaction, and fine and gross motor skills may be seen over time, associated with an improvement in the communicative abilities and verbal comprehension, and a decreased occurrence of withdrawal behavior (personal observation).

## Abbreviations

(AS): Angelman syndrome; (PWS): Prader-Willi syndrome; (ESACs): extra structurally abnormal chromosomes; (PWS/ASCR): PWS/AS critical region; (FISH): fluorescence *in situ *hybridization; (aCGH): array comparative genomic hybridization; (DSM IV): Diagnostic and Statistical Manual – Revision 4; (EEG): electroencephalogram; (CT/MRI): computerized tomography/magnetic resonance imaging; (BP): breakpoints; (GABA): gamma-aminobutyric acid; (CNS): central nervous system; (CVS): chorionic villus sampling; (VPA): valproate; (CBZ): carbamazepine; (LTG): lamotrigine.

## Competing interests

The author declares that he has no competing interests.

## References

[B1] Webb T (1994). Inv dup (15) supernumerary marker chromosomes. J Med Genet.

[B2] Webb T, Hardy CA, King M, Watkiss E, Mitchell C, Cole T (1998). A clinical, cytogenetic and molecular study of ten probands with inv dup (15) marker chromosomes. Clin Genet.

[B3] Schinzel A, Niedrist D (2001). Chromosome imbalances associated with epilepsy. Am J Med Genet (Semin Med Genet).

[B4] Wang NJ, Liu D, Parokonny AS, Schanen NC (2004). High-resolution molecular characterization of 15q11-q13 rearrangements by array comparative genomic hybridization (array CGH) with detection of gene dosage. Am J Hum genet.

[B5] Dennis NR, Veltman MWM, Thompson R, Craig E, Bolton PF, Thomas NS (2006). Clinical findings in 33 subjects with large supernumerary marker(15) chromosomes and 3 subjects with triplication of 15q11-q13. AM J Med Genet.

[B6] Donlon TA, Lalande M, Wyman A, Bruns G, Latt SA (1986). Isolation of molecular probes associated with the chromosome 15 instability in the Prader-Willi syndrome. Proc Natl Acad Sci USA.

[B7] Christian SL, Fantes JA, Mewborn SK, Huang B, Ledbetter D (1999). Large genomic duplicons map to sites of instability in the Prader-Willi/Angelman syndrome chromosome region (15q11-q13). Hum Mol Genet.

[B8] Makoff AJ, Flomen RH (2007). Detailed analysis of 15q11-q14 sequence corrects errors and gaps in the public access sequence to fully reveal large segmental duplications at breakpoints for Prader-Willi, Angelman, and inv dup(15) syndromes. Genome Biol.

[B9] Lalande M (1996). Parental imprinting and human disease. Ann Rev Genet.

[B10] Browne CE, Dennis NR, Maher E, Long FL, Nicholson JC, Sillibourne J, Barber JC (1997). Inherited interstitial duplications of proximal 15q: genotype-phenotype correlations. Am J Hum Genet.

[B11] Maraschio P, Cuococ C, Gimelli G, Zuffardi O, Tiepolo L, Danil A (1988). Origin and clinical significance of inv dup (15). The cytogenetics of mammalian autosomal rearrangements.

[B12] Leana-Cox J, Jenkins L, Palmer CG, Plattner R, Sheppard L, Flejter WL, Zackowski J, Tsien F, Schwartz S (1994). Molecular cytogenetic analysis of inv dup (15) chromosomes, using probes specific for the Prader-Willi/Angelman syndrome region: clinical implications. Am J Hum Genet.

[B13] Crolla JA, Harvey JF, Sitch FL, Dennis NR (1995). Supernumerary marker 15 chromosomes: a clinical, molecular and FISH approach to diagnosis and prognosis. Hum Genet.

[B14] Huang B, Crolla JA, Christian SL, Wolf-Ledbetter ME, Macha ME, Papenhausen PN, Ledbetter DH (1997). Refined molecular characterization of the breakpoints in small inv dup (15) chromosomes. Hum Genet.

[B15] Cheng SD, Spinner NB, Zackai EH, Knoll JH (1994). Cytogenetic and molecular characterization of inverted duplicated chromosomes 15 from 11 patients. Am J Hum Genet.

[B16] Hou JW, Wang TR (1998). Unusual features in children with inv dup(15) supernumerary marker: a study of genotype-phenotype correlation in Taiwan. Eur J Pediatr.

[B17] Robinson WP, Binkert F, Giné R, Vazquez C, Müller W, Rosenkranz W, Schinzel A (1993). Clinical and molecular analysis of five inv dup (15) patients. Eur J Hum Genet.

[B18] Blennow E, Nielsen KB, Telenius H, Carter NP, Kristoffersson U, Holmberg E, Gillberg C, Nordenskjöld M (1995). Fifty probands with extra structurally abnormal chromosomes characterized by fluorescence in situ hybridization. Am J Med Genet.

[B19] Wang NJ, Parokonny AS, Thatcher KN, Driscoll J, Malone BM, Dorrani N, Sigman M, LaSalle JM, Schanen NC (2008). Multiple forms of atypical rearrangements generating supernumerary derivative chromosome 15. BMC Genetics.

[B20] Flejter WL, Bennet-Barker PE, Ghaziuddin M, McDonald M, Sheldon S, Gorski JL (1996). Cytogenetic and molecular analysis of inv dup(15) chromosomes observed in two patients with autistic disorder and mental retardation. Am J Med Genet.

[B21] Battaglia A, Gurrieri F, Bertini E, Bellacosa A, Pomponi MG, Paravatou-Petsotas M, Mazza S, Neri G (1997). The inv dup(15) syndrome: a clinically recognizable syndrome with altered behaviour, mental retardation and epilepsy. Neurology.

[B22] Luke S, Verma RS, Giridharan R, Conte RA, Macera MJ (1994). Two Prader-Willi/Angelman syndrome loci present in an isodicentric marker chromosome. Am J Med Genet.

[B23] Crolla JA, Youings SA, Ennis S, Jacobs PA (2005). Supernumerary marker chromosomes in man: parental origin, mosaicism and maternal age revisited. Eur J Hum Genet.

[B24] Loitzsch A, Bartsch O (2006). Healthy 12-year-old boy with mosaic inv dup(15)(q13). Am J Med Genet.

[B25] Saitoh S, Hosoki K, Takano K, Tonoki H (2007). Mosaic paternally derived inv dup(15) may partially rescue the Prader-Willi syndrome phenotype with uniparental disomy. Clin Genet.

[B26] Gillberg C, Steffenburg S, Wahlström J, Gillberg IC, Sjöstedt A, Martinsson T, Liedgren S, Eeg-Olofsson O (1991). Autism associated with marker chromosome. J Am Acad Child Adolesc Psychiat.

[B27] Battaglia A (2005). The inv dup(15) or idic(15) syndrome: a clinically recognisable neurogenetic disorder. Brain Devel.

[B28] Rineer S, Finucane B, Simon EW (1998). Autistic symptoms among children and young adults with isodicentric chromosome 15. Am J Med Genet.

[B29] Borgatti R, Piccinelli P, Passoni D, Dalprà L, Miozzo M, Micheli R, Gagliardi C, Balottin U (2001). Relationship between clinical and genetic features in "inverted duplicated chromosome 15" patients. Pediatr Neurol.

[B30] Bingham PM, Spinner NB, Sovinsky L, Zackai EH, Chance PF (1996). Infantile spasms associated with proximal duplication of chromosome 15q. Pediatr Neurol.

[B31] Mignon C, Malzac P, Moncla A, Depetris D, Roeckel N, Croquette MF, Mattei MG (1996). Clinical heterogeneity in 16 patients with inv dup (15) chromosome: cytogenetic and molecular studies, search for an imprinting effect. Eur J hum Genet.

[B32] Aguglia U, Le Piane E, Gambardella A, Messina D, Russo C, Sirchia SM, Porta G, Quattrone A (1999). Emotion-induced myoclonic absence-like seizures in a patient with inv-dup(15) syndrome: a clinical, EEG, and molecular genetic study. Epilepsia.

[B33] Chifari R, Guerrini R, Pierluigi M, Cavani S, Sgrò V, Elia M, Canger R, Canevini MP (2002). Mild generalized epilepsy and developmental disorder associated with large inv dup (15). Epilepsia.

[B34] Gobbi G, Genton P, Pini A, Gurrieri F, Livet MO, Roger J, Bureau M, Dravet Ch, Genton P, Tassinari CA, Wolf P (2002). Epilepsies and chromosomal disorders. Epileptic syndromes in infancy, childhood and adolescence.

[B35] Centerwall WR, Morris JP (1975). Partial D15 trisomy. A case and general review. Hum Hered.

[B36] Schreck RR, Breg WR, Erlanger BF, Miller OJ (1977). Preferential derivation of abnormal human G-group-like chromosomes from chromosome 15. Hum Genet.

[B37] Schinzel A, Schmid W, Nielsen J (1981). Particular behavioural symptomatology in patients with rarer autosomal chromosome aberrations. Human behavior and genetics.

[B38] Qumsiyeh MB, Rafi SK, Sarri C, Grigoriadou M, Gyftodimou J, Pandelia E, Laskari H, Petersen MB (2003). Double supernumerary isodicentric chromosomes derived from 15 resulting in partial hexasomy. Am J Med Genet.

[B39] Schinzel A (2001). Catalogue of unbalanced chromosome aberrations in man.

[B40] Grosso S, Balestri P, Anichini C, Bartalini G, Pucci L, Morgese G, Berardi R (2001). Pubertal disorders in inv dup(15) syndrome. Gynecol Endocrinol.

[B41] Connor JM, Gilmore DH (1984). An analysis of the parental age effect for inv dup (15). J Med Genet.

[B42] Schinzel A, Brecevic L, Bernasconi F, Binkert F, Berthet F, Wuilloud A, Robinson WP (1994). Intrachromosomal triplication of 15q11-q13. J Med Genet.

[B43] Dittrich B, Buiting K, Korn B, Rickard S, Buxton J, Saitoh S, Nicholls RD, Poustka A, Winterpacht A, Zabel B, Horsthemke B (1996). Imprint swithing on human chromosome 15 may involve alternative transcripts of the SNRPN gene. Nat Genet.

[B44] Mohandas TK, Park JP, Spellman RA, Filiano JJ, Mamourian AC, Hawk AB, Belloni DR, Noll WW, Moeschler JB (1999). Paternally derived de novo interstitial duplication of proximal 15q in a patient with developmental delay. Am J Med Genet.

[B45] Nakatsu Y, Tyndale RF, DeLorey TM, Durham-Pierre D, Gardner JM, McDanel HJ, Nguyen Q, Wagstaff J, Lalande M, Sikela JM (1993). A cluster of three GABAA receptor subunit genes is deleted in a neurological mutant of the mouse p locus. Nature.

[B46] Cook EH, Courchesne RY, Cox NJ, Lord C, Gonen D, Guter SJ, Lincoln A, Nix K, Haas R, Leventhal BL, Courchesne E (1998). Linkage-disequilibrium mapping of autistic disorder, with 15q11-13 markers. Am J Hum Genet.

[B47] Caron L, Rousseau F, Gagnon E, Isenring P (2000). Cloning and functional characterization of a cation-Cl cotransporter-interacting protein. J Biol Chem.

[B48] Hogart A, Leung KN, Wang NJ, Wu DJ, Driscoll J, Vallero RO, Schanen NC, LaSalle JM Chromosome 15q11-13 duplication sindrome brain reveals epigenetic alterations in gene expression not predicted from copy number. J Med Genet.

[B49] Nietzel A, Albrecht B, Starke H, Heller A, Gillesen-Kaesbach G, Claussen U, Liehr T (2003). Partial hexasomy 15pter→ 15q13 including SNRPN and D15S10: first molecular cytogenetically proven case report. J Med Genet.

[B50] Miny P, Basaran S, Kuwertz E, Holzgreve W, Pawlowitzki IH (1986). Inv dup (15): prenatal diagnosis and postnatal follow-up. Prenat Diagn.

[B51] (2008). IDEAS Physician Advisory. http://www.idic15.org.

